# Novel Antidepressant-Like Activity of Propolis Extract Mediated by Enhanced Glucocorticoid Receptor Function in the Hippocampus

**DOI:** 10.1155/2013/217853

**Published:** 2013-06-19

**Authors:** Mi-Sook Lee, Young Han Kim, Wan-Soon Park, Won Gyeong Ahn, Ok Kyu Park, Seung-Hae Kwon, Kyoji Morita, Insop Shim, Song Her

**Affiliations:** ^1^Division of Bio-Imaging, Chuncheon Center, Korea Basic Science Institute, Chuncheon 200-701, Republic of Korea; ^2^Acupuncture & Meridian Science Research Center, College of Oriental Medicine, Kyung Hee University, Seoul 130-701, Republic of Korea; ^3^Laboratory of Neuropharmacology, Department of Nursing, Shikoku University, School of Health Sciences, Tokushima 771-1192, Japan

## Abstract

Propolis is a natural product made by honeybees that has been widely used in folk medicine with a broad spectrum of biological activities. To investigate the antidepressant-like activity of propolis extract, CD-1 mice were administered an ethanol extract of propolis (50, 100, or 200 mg/kg, p.o.) prior to the behavioral test. The propolis extract-treated group showed a dose-dependent decrease in immobility time in the FST and tail suspension test without altering locomotor activity. Propolis extract decreased the limbic hypothalamic-pituitary-adrenal axis response to the FST as indicated by an attenuated corticosterone response and decreased in c-fos immunoreactive neurons in the hippocampal dentate gyrus. Western blot analysis revealed a reduction in hippocampal glucocorticoid receptor (GR) expression following the FST, which was reversed by propolis extract. Propolis extract also increased pGR(S220)/(S234) ratio by a differential phosphorylation in S220 and S234. FST-induced downregulation of cAMP-responsive element binding protein phosphorylation at S133 (pCREB) was restored by propolis extract, showing a strong and positive relationship between pCREB and pGR(S220)/(S234) ratio. These findings suggest that the propolis extract potentiates antidepressant-like activity by enhancing GR function which is one of the therapeutic mechanisms of antidepressant; thus, propolis extract may provide a novel therapy for depression.

## 1. Introduction 

Propolis is a resinous substance collected by honeybees from various plants. It contains more than 200 natural constituents including polyphenols, phenolic aldehydes, sequiterpene-quinones, coumarins, amino acids, fatty acids, steroids, and inorganic compounds [1]. Propolis has been extensively used in health care products and pharmaceutical preparations [2]. The pharmacological properties of propolis exhibit antimicrobial, antioxidant, anti-inflammatory, antitumor, and neuroprotective activities *in vitro* and *in vivo *[3–7]. Moreover, recent studies have shown that propolis essential oil has an anxiolytic effect on an acutely stressed mouse model through modulation of the hypothalamic-pituitary-adrenal (HPA) axis [8, 9]. This finding suggests that propolis has antidepressant-like effect, but this role of the substance has not been investigated. 

 Chronically high glucocorticoid levels in patients with depression are commonly attributed to impaired feedback regulation of the HPA axis [10, 11], possibly caused by impaired glucocorticoid receptor (GR) function in the hippocampus, hypothalamus, and pituitary gland [12]. Human postmortem studies have shown that GR expression (mRNA and protein) is downregulated in suicide victims with a history of childhood abuse [11, 13, 14]. Furthermore, several studies have shown that antidepressants restored GR function by increasing GR expression and thereby alleviate depressive symptoms [15–17]. These findings indicate that restoration of GR function is an important component of the antidepressant therapeutic action for depression.

GR function is affected by the posttranslational GR modification, including phosphorylation [18, 19]. The most highly phosphorylated residues in the human GR N-terminus are S203, S211, and S226 (corresponding to S212, S220, and S234 in mice) [18]. In particular, ligand-dependent phosphorylation at S211 (pGR(S211)) enhances the GR transcriptional response, whereas phosphorylation at S226 (pGR(S226)) reduces nuclear retention of GR, leading to attenuation of GR transcriptional activity [20, 21]. Thus, the level and/or ratio of pGR(S211) and pGR(S226) may reflect GR activity with respect to stress-related disorders. In line with this, we previously reported that Cortex Mori Radicis extract increased the pGR(S211)/(S226) ratio in the hippocampus of rats, which enhances GR function, and thereby facilitates inhibition of limbic HPA feedback [22]. 

In the present study, we report an antidepressant-like activity of ethanol extract of Korean propolis in the forced swim test (FST) and tail suspension test (TST). To gain further insight into the neurobiological effects of propolis extract on antidepressant-like activity, the effect of the extract on corticosterone response and c-fos immunoreactivity was evaluated in mice exposed to the FST. Furthermore, we investigated the molecular mechanism underlying the antidepressant-like activity of propolis by assessing changes in phosphorylation status of GR and CREB, both of which play key roles in stress-related modulation of glucocorticoids in the HPA axis [23, 24].

## 2. Materials and Methods 

### 2.1. Animals

Four-week-old male CD-1 mice (Orient Co., Seoul, Korea) were housed five per cage in a temperature-controlled (22–24°C) room with a 12 h light/dark cycle. The lights were on from 8:00 to 20:00. The mice were allowed at least one week to acclimate to the environment before the experiments commenced. The Institutional Animal Care and Use Committee at the Korea Basic Science Institute (KBSI) reviewed and approved the present study (KBSI-AEC 1202). All animal procedures were conducted in accordance with the “Guide for the Care and Use of Laboratory Animals” issued by the Laboratory Animal Resources Commission of KBSI.

### 2.2. Compound Administration and Experimental Procedure

Ethanol extract (95%, w/v) prepared from Korean propolis was purchased from Milseong Bee Farm (Yeoju, Korea) where propolis was collected by honeybees between June and October in the Kangwon region of Korea. Fluoxetine, kindly provided by Daewoo Pharmaceuticals (Busan, Korea), was dissolved in saline and used as a positive control for antidepressant activity. 

The experimental conditions included three doses of propolis extract (50, 100, or 200 mg/kg, p.o., *n* = 10 per group), fluoxetine (10 mg/kg, p.o., *n* = 10), and a vehicle group (saline, 1 mL/kg, p.o., *n* = 10). The animals were pre-treated with compound or saline three times at 24, 12, and 1 h prior to the FST. Thirty minutes after the FST procedure, the animals were scarified for hormone assay, western blot analysis and immunohistochemistry in which a group of 10 mice not exposed to FST served as controls (naive). 

### 2.3. Forced Swim Test

The modified FST described by Porsolt and colleagues [25] was used in the present study. Briefly, mice were forced to swim for 5 min in a glass beaker (diameter, 13 cm; height, 19 cm) filled to a depth of 14 cm with 23–25°C water. Behavioral responses (i.e., immobility, climbing, and swimming) were videotaped during the 5 min test session and analyzed by two trained observers who were blind to the treatment.

### 2.4. Tail Suspension Test

An independent subset of 10 mice was used for the TST following administration of propolis extract or fluoxetine [26]. In brief, each mouse was suspended on the edge of a lever 58 cm above a tabletop using adhesive tape placed approximately 1 cm from the tip of the tail. A mouse was considered immobile when it hung passively and was completely motionless. The total duration of immobility was measured over a 6 min period by blinded observers.

### 2.5. Open-Field Test

After the TST, ambulatory behavior was examined in an open-field test as described previously [27]. The apparatus consisted of a black Plexiglas square (45 × 45 × 40 cm) with visible lines dividing the floor into nine 15 × 15 cm squares drawn using a marker. Mice were individually placed into the center of the apparatus and allowed to explore freely. A blinded observer scored the number of times a mouse crossed one of the grid lines with all four paws during a 10 min observation period. 

### 2.6. Corticosterone Assay

The corticosterone assay has been described previously [22]. Briefly, trunk blood was rapidly collected 30 min after onset of the FST. Corticosterone serum levels were measured using a commercially available enzyme immunoassay (EIA) kit (R&D Systems, Inc., Minneapolis, MN, USA), according the manufacturer's instructions. Trunk blood from animals not subjected to the FST was obtained and analyzed using the same procedure.

### 2.7. Immunohistochemical Analysis

Mice (*n* = 10 per group) were sacrificed following the FST, and the brains were collected and fixed overnight in freshly prepared 4% paraformaldehyde in phosphate-buffered saline (PBS). Immunofluorescent staining was performed on 20 *μ*m sections using antibodies specific for c-fos (1 : 500; Santa Cruz Biotechnology, Santa Cruz, CA, USA), phospho-CREB (S133) (1 : 500; Cell Signaling Technology, Danvers, MA, USA), or NeuN (1 : 500; EMD Millipore Corporation, Billerica, MA, USA) and subsequently exposed to Alexa 488-labelled goat anti-rabbit antibody (1 : 500; Invitrogen, Paisley, UK) or Alexa 546-labelled goat anti-mouse antibody (1 : 500; Invitrogen). Sections were counterstained with DRAQ-5 (1 : 3,000; Biostatus Limited, Shepshed, Leicestershire, UK). Fluorescence signals were visualized using a confocal laser scanning microscope (LSM 780 NLO; Carl Zeiss, Oberkochen, Germany), and a serial z-stack (1 *μ*m step size) of 4–6 confocal images was flattened into a single two-dimensional image using LSM image analysis software (ver. 4.2; Carl Zeiss). The number of c-fos immunoreactive neurons in the entire dentate gyrus (DG) granule cell layer was counted by a blinded observer. We analyzed all granule cells in four consecutive sections of the DG of each animal between 1.46 and 2.18 mm anterior to Bregma according to the Paxinos and Watson atlas [28]. The average cell count was expressed as the number of c-fos-positive neurons per area (mm^2^).

### 2.8. Western Blot Analysis

The western blot analysis of extracts of frozen hippocampal tissue was carried out as described previously [29]. Protein concentrations were determined using a bicinchoninic acid (BCA) protein assay (Pierce Biotechnology, Rockford, IL, USA). The primary antibodies were specific for GR (1 : 1,000; Santa Cruz Biotechnology), phospho-GR (S220) (1 : 500; Cell Signaling Technology), phospho-GR (S234) (1 : 500; Abcam, Cambridge, UK), CREB (1 : 500; Cell Signaling Technology), phospho-CREB (S133) (1 : 500; Cell Signaling Technology), and *β*-actin (1 : 10,000; Sigma, St. Louis, MO, USA). Detection was carried out using horseradish peroxidase-conjugated IgG (1 : 5,000; Santa Cruz Biotechnology) and visualized using an electrochemiluminescence (ECL) assay kit (Amersham, Little Chalfont, Buckinghamshire, UK). The band intensities obtained by western blot analysis were determined using the ImageJ program (open source ImageJ software available at http://rsb.info.nih.gov/ij/).

### 2.9. Statistical Analysis

Data were analyzed using a one-way analysis of variance (ANOVA), followed by the Tukey *post hoc* test using Prism 4 (GraphPad Software, Inc., San Diego, CA, USA) for multigroup comparisons. Pearson's correlation coefficients were calculated for each pGR(S220)/(S234) ratio and pCREB/CREB to detect statistical correlations. The level of statistical significance was deemed to be *P* < 0.05. Results are expressed as mean ± standard errors of the mean (SEM). 

## 3. Results 

### 3.1. Antidepressant-Like Behavioral Effect of Propolis Extract

**Figure 1:**
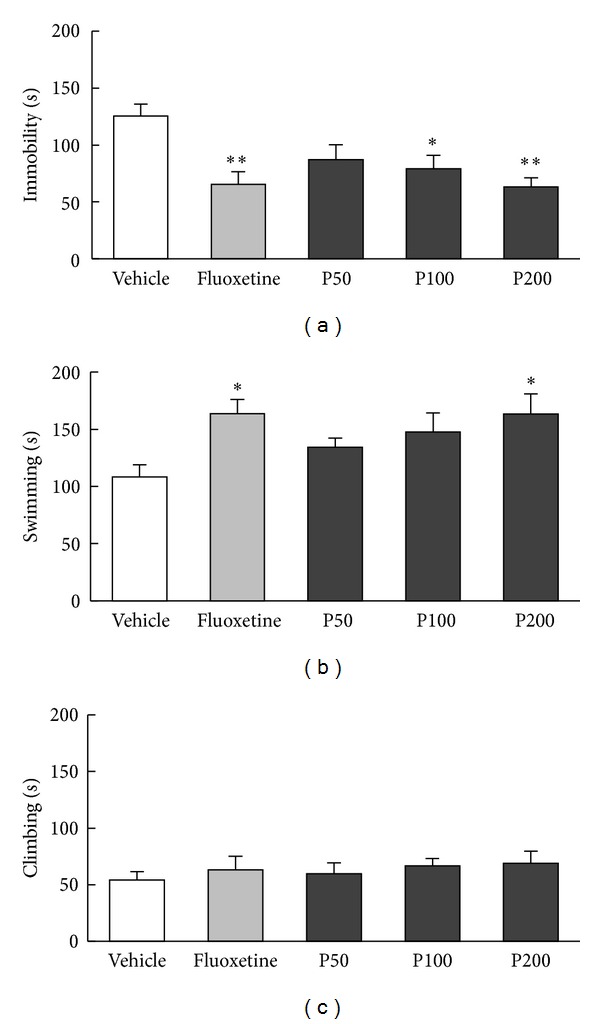
Effects of propolis extract on depressive-like behaviors during the FST. Vehicle (1 mL/kg saline), propolis extract (50, 100, or 200 mg/kg), or fluoxetine (10 mg/kg) was orally administered 24, 12, and 1 h prior to FST procedure. Immobility (a), swimming (b), and climbing time (c) were recorded during a 5 min test session. The columns and error bars represent means ± SEM (*n* = 10 per group). Data were analyzed using a one-way ANOVA followed by Tukey's* post hoc* test. P50: propolis extract (50 mg/kg); P100: propolis extract (100 mg/kg); P200: propolis extract (200 mg/kg). *P < 0.05, **P < 0.01 versus vehicle.

A significant effect of treatment was found for the FST-induced behaviors of immobility time (Figure 1(a), *F*_4,45_ = 5.15, *P* < 0.01) and swimming (Figure 1(b), *F*_4,45_ = 2.96, *P* < 0.05). However, no significant difference among groups was found for climbing behavior (Figure 1(c), *F*_4,45_ = 0.38, *P* = 0.82). The *post hoc* test revealed that propolis extract (100 and 200 mg/kg) decreased immobility time during the FST in a dose-dependent manner compared with vehicle treatment (*P* < 0.05; *P* < 0.01, resp.). Furthermore, propolis (200 mg/kg) significantly increased swimming time (*P* < 0.05 versus vehicle). Fluoxetine (10 mg/kg), used as a positive control, markedly decreased immobility time and increased swimming time compared with vehicle treatment during the FST (*P* < 0.01; *P* < 0.05, resp.). 

**Figure 2:**
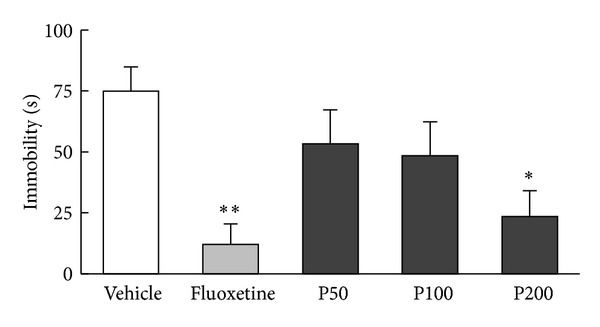
Effects of propolis extract on depressive-like behaviors during the TST. Vehicle (1 mL/kg saline), propolis extract (50, 100, or 200 mg/kg), or fluoxetine (10 mg/kg) was orally administered 24, 12, and 1 h prior to TST procedure. Immobility time was recorded during a 6 min test session. The columns and error bars represent means ± SEM (*n* = 10 per group). Data were analyzed using one-way ANOVA followed by Tukey's *post hoc* test. P50: propolis extract (50 mg/kg); P100: propolis extract (100 mg/kg); P200: propolis extract (200 mg/kg). *P < 0.05, **P < 0.01 versus vehicle.

**Figure 3:**
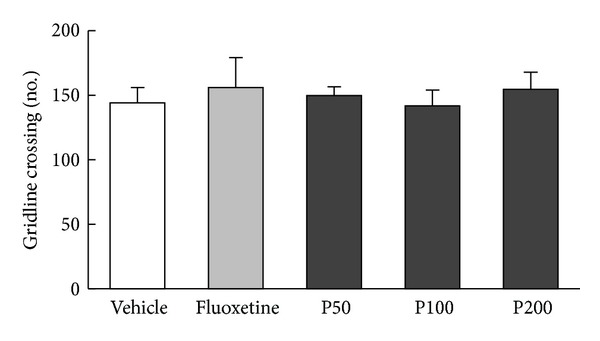
Effects of propolis extract on locomotor activity in the open-field test. After the TST, the spontaneous motor activity of mice was measured by the open-field test. The number of times a gridline was crossed with all four paws was scored during the 10 min test period. The error bars represent the means ± SEM (*n* = 10 per group). Data were analyzed using one-way ANOVA followed by Tukey's *post hoc* tests. The columns and error bars represent means ± SEM (*n* = 10 per group). Data were analyzed using a one-way ANOVA followed by Tukey's *post hoc* test. P50: propolis extract (50 mg/kg); P100: propolis extract (100 mg/kg); P200: propolis extract (200 mg/kg).

A significant effect of treatment was found for immobility time during the TST (Figure 2, *F*_4,45_ = 4.68, *P* < 0.01). The *post hoc* test revealed that the propolis extract (200 mg/kg) and fluoxetine treatments significantly reduced immobility time compared with the vehicle treatment (*P* < 0.05; *P* < 0.01, resp.). However, neither propolis extract nor fluoxetine had a significant effect on the number of grid lines crossed in the open-field test (Figure 3, *F*_4,45_ = 0.02, *P* = 0.99), indicating that locomotor activity was not altered by propolis extract or fluoxetine treatment.

### 3.2. c-fos Response to Propolis Extract in the DG

**Figure 4:**
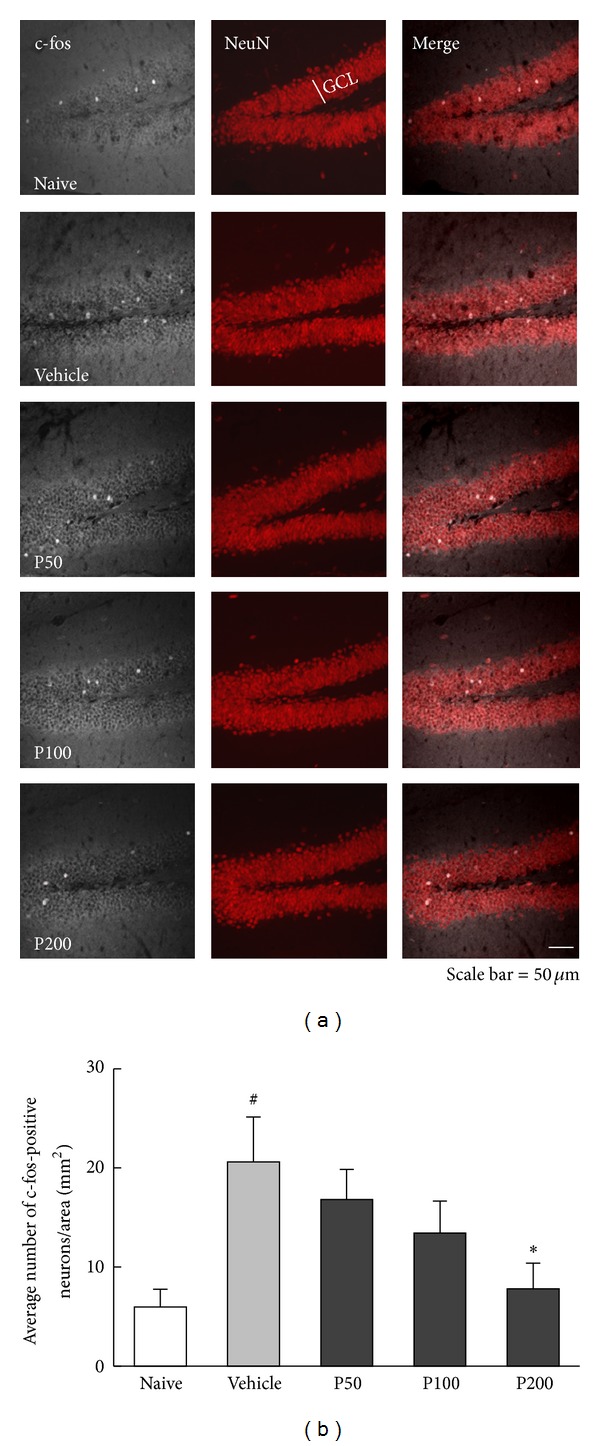
Effect of propolis extract on FST-induced c-fos expression in the hippocampal DG. (a) Representative confocal images of c-fos-positive neurons in the granular cell layer of the DG in mice administered vehicle (1 mL/kg saline), propolis extract (50, 100, or 200 mg/kg). The no FST (naive) group served as the control group. Frozen hippocampal sections were double immunostained with anti-c-fos (white) and anti-NeuN (red). Scale bar = 50 *μ*m. (b) The average number of c-fos-positive neurons in the granular cell layer of the DG. The columns and error bars represent means ± SEM (*n* = 10 per group). Data were analyzed using a one-way ANOVA followed by Tukey's *post hoc* test. GCL: granular cell layer; P50: propolis extract (50 mg/kg); P100: propolis extract (100 mg/kg); P200: propolis extract (200 mg/kg). ^#^ *P* < 0.05 versus naive; *P < 0.05 versus vehicle.

The effect of propolis extract on the neuronal response to the FST was assessed by counting c-fos-positive neurons in the hippocampal DG (Figure 4(a)). The results revealed a significant effect of propolis extract treatment on c-fos expression 30 min after the FST (Figure 4(b), *F*_4,45_ = 3.72, *P* < 0.05) such that the FST-induced increase in c-fos expression (*P* < 0.05 versus naive) was significantly reduced by propolis extract treatment (200 mg/kg, *P* < 0.05 versus vehicle) in a dose-dependent manner. The number of c-fos-positive neurons was low in the hippocampal CA1 and CA2/CA3 subfields (1-2 cells/section, data not shown). 

### 3.3. Propolis Extract-Induced Downregulation of Corticosterone

**Figure 5:**
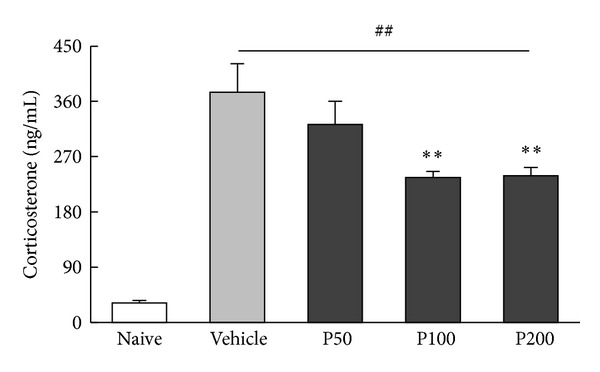
Effect of propolis extract treatment on serum corticosterone levels in mice exposed to the FST. Trunk blood was collected 30 min after the FST. The no FST (naive) group served as the control group. The columns and error bars represent means ± SEM (*n* = 10 per group). Data were analyzed using a one-way ANOVA followed by Tukey's *post hoc* test. P50: propolis extract (50 mg/kg); P100: propolis extract (100 mg/kg); P200: propolis extract (200 mg/kg). ^##^ *P* < 0.01 versus naive; **P < 0.01 versus vehicle.

As shown in Figure 5, serum corticosterone levels were significantly different among the treatment groups (*F*_4,45_ = 22.46, *P* < 0.01). Elevated serum corticosterone levels in vehicle-treated mice exposed to the FST (*P* < 0.01 versus naive) were attenuated by propolis extract treatment (100 and 200 mg/kg, both *P* < 0.01 versus vehicle). 

### 3.4. Modulatory Effect of Propolis Extract on GR Expression and Phosphorylation in the Hippocampus

**Figure 6:**
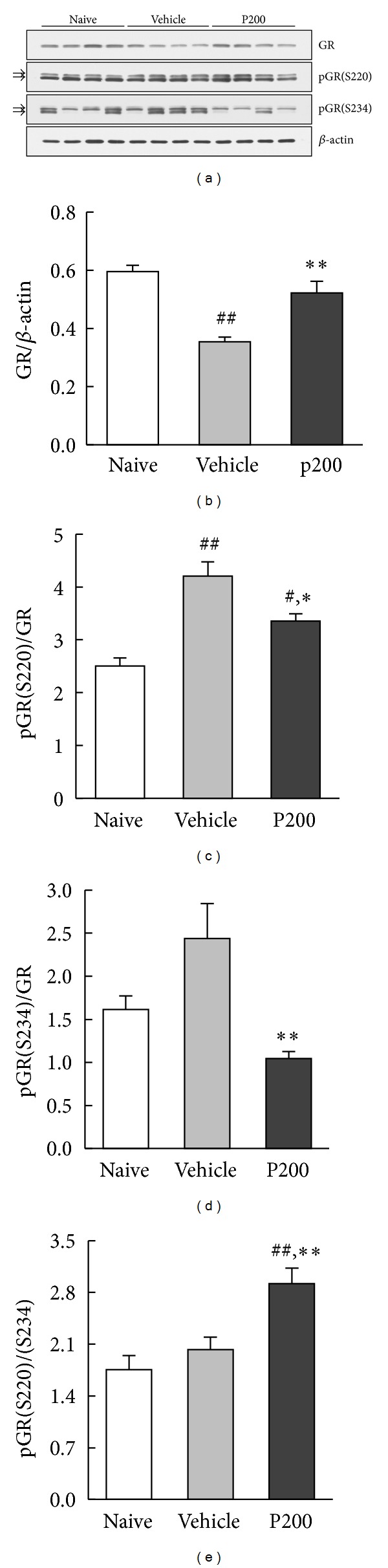
Differential regulation of GR phosphorylation by propolis extract. Following the FST procedure, the hippocampus was dissected and frozen on dry ice. The no FST (naive) group was used as a control group. (a) Representative western blot images showing the expression of pGR(S220), pGR(S234), GR, and *β*-actin from hippocampal lysates. Average band intensity of (b) GR, (c) pGR(S232), (d) pGR(S234), and (e) pGR(S220)/(S234) ratio. GR protein was normalized by *β*-actin. pGR(S220) and pGR(S234) were normalized by total GR protein. The columns and error bars represent means ± SEM (*n* = 10 per group). Data were analyzed using a one-way ANOVA followed by Tukey's *post hoc* test. P200: propolis extract (200 mg/kg). ^#^ *P* < 0.05, ^##^ *P* < 0.01 versus naive; *P < 0.05, **P < 0.01 versus vehicle.

The effect of propolis extract (200 mg/kg) on GR function was assessed by changes of GR protein in whole hippocampal lysates. As shown in Figure 6, a significant effect of treatment on hippocampal GR expression was found (Figure 6(b), *F*_2,27_ = 19.31, *P* < 0.01). Exposure to the FST significantly decreased hippocampal GR expression in the vehicle-treated group (*P* < 0.01 versus naive), which was restored by propolis extract treatment (*P* < 0.01 versus vehicle). We then investigated the phosphorylation status of GR at S220 and S234, sites known to play a key role in the regulation of GR transcriptional activity [30]. Two bands of phosphorylated forms were detected in all samples, perhaps towing to different patterns of expression of phosphorylated isoforms carrying other posttranslational modifications. A significant effect of propolis extract treatment was found for both phosphorylation sites in the hippocampal GR (Figure 6(c), *F*_2,27_ = 18.61, *P* < 0.01 for pGR(S220); Figure 6(d), *F*_2,27_ = 7.56, *P* < 0.01 for pGR(S234)). pGR(S220) levels, which were significantly increased in vehicle-treated mice exposed to the FST (*P* < 0.01 versus naive), were significantly attenuated by propolis extract treatment (*P* < 0.05 versus vehicle); however, pGR(S220) levels in the propolis extract treatment group remained higher than those of the naive group (*P* < 0.05 versus naive). In contrast, propolis extract significantly decreased pGR(S234) levels compared with those of the vehicle group (*P* < 0.01), suggesting a differential phosphorylation between these two sites. The pGR(S220)/(S234) ratio was calculated to quantitatively compare GR phosphorylation at S220 and S234. A significant effect of treatment was found for pGR(S220)/(S234) ratio (Figure 6(e), *F*_2,27_ = 10.19, *P* < 0.01) such that the ratio of the propolis extract-treated group was significantly higher than those of the naive and vehicle groups (*P* < 0.01 versus naive; *P* < 0.01 versus vehicle), indicating increased GR function. These results suggest that propolis extract enhanced GR function via the additive actions of increased GR expression and pGR(S220)/(S234) ratio.

### 3.5. Enhanced CREB Phosphorylation and the Relationship between pCREB and pGR(S220)/(S234) Ratio

**Figure 7:**
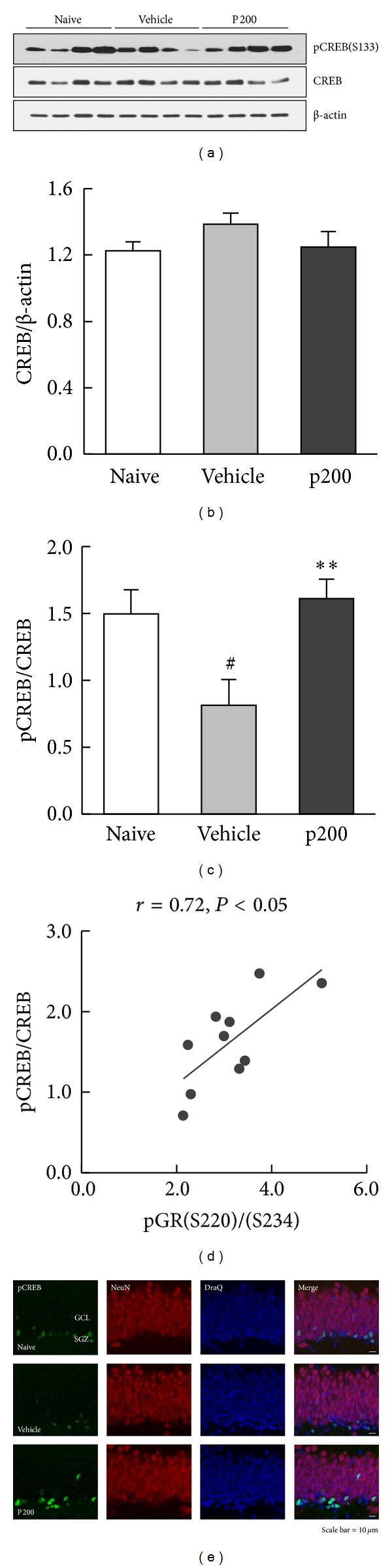
Effect of propolis extract on CREB phosphorylation in the hippocampus. The no FST (naive) group served as the control group. (a) Representative western blot images showing the expression of pCREB, CREB, and *β*-actin in hippocampal lysates. Average band intensity of (b) CREB and (c) pCREB/CREB ratio. CREB and pCREB protein were normalized by *β*-actin and CREB, respectively. The columns and error bars represent means ± SEM (*n* = 10 per group). Data were analyzed using a one-way ANOVA followed by Tukey's *post hoc* test. P200, propolis extract (200 mg/kg). ^#^ *P* < 0.05 versus naive; **P < 0.01 versus vehicle. (d) Correlation between the pCREB/CREB and pGR(S220)/(S234) ratio. The lines represent the linear data fit. Pearson's correlation coefficient (*r*) is shown above the plot (*n* = 10). (e) Representative immunofluorescence images of pCREB in the hippocampal DG. Frozen hippocampal sections were triple-immunostained with anti-pCREB (green) and anti-NeuN (red) followed by DraQ (blue) nuclear counterstaining. It is apparent that pCREB protein is enriched in the nucleus of cells in the DG region. Scale bar = 10 *μ*m. GCL: granular cell layer; SGZ: subgranular zone.

cAMP response element-binding protein (CREB) is thought to be a convergence point for antidepressant drugs [31]; thus, we used western blot analysis to examine the expression of CREB and its phosphorylation status at S133 (Figure 7(a)). CREB expression in hippocampal lysates did not differ among the groups (Figure 7(b), *F*_2,27_ = 1.39, *P* = 0.27); however, a significant treatment effect was found for pCREB (Figure 7(c), *F*_2,27_ = 6.14, *P* < 0.01). Exposure to the FST significantly reduced pCREB in vehicle-treated animals (*P* < 0.05 versus naive), which was restored by propolis extract treatment (*P* < 0.01 versus vehicle). CREB activation by phosphorylation was confirmed using immunofluorescence in which pCREB immunoreactivity was elevated in propolis extract-treated slices compared with vehicle-treated slices of hippocampal DG (Figure 7(e)). Moreover, a strong positive linear correlation was found between pCREB and the pGR(S220)/(S234) ratio (Figure 7(d), *r* = 0.72, *P* < 0.01). These data suggest that CREB activation via phosphorylation at S133 is significantly correlated with GR function in response to propolis extract. 

## 4. Discussion

The results of the present paper indicate that propolis extract has antidepressant-like activity in mice exposed to the FST mediated by an increase in GR function as a result of additive modulation of increased GR expression and phosphorylation. Furthermore, our findings indicate that increased GR function is functionally linked to CREB signaling. 

We found an antidepressant-like activity in CD-1 mice administered fluoxetine three times at 24, 12, and 1 h prior to the FST. This finding is consistent with that of previous studies [32, 33] and confirms that our animal model was suitable for the investigation of an antidepressant effect of propolis extract. Using the same experimental protocol, we showed that propolis extract had a dose-dependent antidepressant-like effect on the FST-induced depressive-like behavior, as demonstrated by reduced immobility time at 100 and 200 mg/kg and increased swimming time at 200 mg/kg. Although the FST is frequently used to screen for antidepressant drugs, the predictive validity of the test alone is not sufficient; thus, we used the TST to confirm the antidepressant-like activity of propolis extract. We found that propolis extract produced a dose-dependent decrease in immobility time during the TST. Antidepressants such as imipramine have been shown to affect locomotor activity, which could lead to false-positive results [34]. We used an open-field test to ensure the positive responses observed that were due to the propolis extract's antidepressant-like effect and not from the extract-induced increase in activity. Our findings of a decrease in immobility time and an increase in active behavior suggest that the propolis extract has antidepressant-like activity without affecting locomotor activity.

A marked suppression of c-fos was found in the hippocampal DG of the propolis extract-treated group. The results of the current study show that exposure to the FST elicited c-fos induction in the hippocampal DG of mice, which was reversed by the administration of propolis extract in a dose-dependent manner. This finding is in agreement with our previous observations using a rat model where c-fos immunoreactivity was decreased by Cortex Mori Radicis extract, exhibiting antidepressant-like activity [22]. Furthermore, downregulated c-fos expressions were also found in the study of antioxidant and anti-inflammatory activities by caffeic acid phenethyl ester (CAPE), an active biomaterial component of propolis [35]. Thus, it is tempting to speculate that CAPE may show antidepressant-like activity. Propolis extract-induced suppression of c-fos is clearly indicative of the complex behavioral responses elicited by the FST and may provide insight into the neural circuits potentially involved in mediating the HPA response to the FST, particularly when compared with neurochemical data. As expected, the HPA axis response to the FST was significantly blunted by propolis extract, resulting in decreased levels of corticosterone. This finding is consistent with previous studies demonstrating that high levels of corticosterone in response to stress are closely correlated with a change in c-fos expression [36] and that antidepressants compensate for impaired feedback inhibition by decreasing neural activity, thereby normalizing HPA activity [37].

The present study sought to demonstrate a molecular link between the antidepressant-like activity of propolis extract and hippocampal GR function in mice subjected to the FST. Previous studies have demonstrated that decreased GR expression associated with major depression was restored by antidepressant treatment [17]. The western blot analysis in our study revealed a similar finding, suggesting that the antidepressant-like activity of propolis extract may be related to GR function in the hippocampus. Evidence for propolis extract enhancement of GR function was further supported by the results of GR phosphorylation status described in previous studies [22]. Although propolis extract significantly decreased pGR(S220) and pGR(S234) levels, the decrease in pGR(S234) was greater than that of pGR(S220), resulting in a high pGR(S220)/(S234) ratio, and thereby enhanced GR function. Differential phosphorylation between these two sites has been reported in patients with major depression in whom peripheral blood mononuclear cells showed an increase in pGR(S226) and to a lesser extent in pGR(S211) compared with control patients [38, 39]. Thus, the increased GR expression and pGR(S220)/(S234) ratio we report here may explain the antidepressant-like activity via enhanced GR function in response to propolis extract treatment.

Studies using rodent models and postmortem tissue have shown that all major classes of antidepressants increase CREB expression and function in several brain regions including hippocampus [40, 41]. Thus, to investigate whether propolis extract affect the CREB expression and/or function, we performed a western blot analysis using antibodies against CREB and pCREB (S133), which is necessary for activation of CREB signaling. Although no changes in CREB expression were found, the FST-induced downregulation of CREB phosphorylation was reversed by treatment with propolis extract. These results were confirmed by pCREB immunostaining, which revealed dense labeling in the hippocampal DG of the propolis extract-treated group, indicating a propolis extract-induced increase in CREB function. Furthermore, the correlation analysis revealed a strong positive linear correlation between pCREB/CREB and pGR(S220)/(S234) ratio (*r* = 0.72). These results suggest that cross talk between GR and CREB signaling may control the gene transcription that underlies the antidepressant-like activity of propolis extract.

 It is interesting to note that the propolis extract-induced pCREB-labeled cells were not colocalized with mature neuron, but were detected primarily in the subgranular zone (SGZ), located between the hilus and granule cell layer, in which newborn neurons typically exist [42]. Increased pCREB expression in the cells around the SGZ suggests that propolis extract may play a role in neurogenesis. This hypothesis is supported by previous studies showing that activation of cAMP-CREB signaling is a critical aspect of neurogenesis under physiological conditions [43]. Moreover, recent studies have reported that CAPE exhibited a neuroprotective effect via brain-derived neurotrophic factor (BDNF), a target gene of CREB signaling and required neurotrophic factor to enhance hippocampal neurogenesis [44, 45]. Thus, further study of propolis extract- or CAPE-induced neurogenesis is warranted.

## 5. Conclusion 

The present study is the first published report to demonstrate an antidepressant-like activity of an ethanol extract of Korean propolis. The antidepressant-like property of the propolis extract may be mediated by the regulation of HPA axis activity via GR signaling correlated with CREB activity. Although further study to identify the active antidepressant components of propolis, including CAPE, is necessary, our findings may provide a novel complementary and alternative treatment for patients with neuropsychiatric disorders, including major depression.
